# Predictive modeling of emergency cesarean delivery

**DOI:** 10.1371/journal.pone.0191248

**Published:** 2018-01-23

**Authors:** Carlos Campillo-Artero, Miquel Serra-Burriel, Andrés Calvo-Pérez

**Affiliations:** 1 Centre for Research in Health and Economics, Universitat Pompeu Fabra, Barcelona, Spain; 2 Balearic Health Service, Palma de Mallorca, Spain; 3 Universitat de Barcelona, Barcelona, Spain; 4 Centre for Research in Health and Economics, Universitat Pompeu Fabra, Barcelona, Spain; 5 Hospital de Manacor, Obstetrics and Gynecology, Carretera Manacor Alcudia, Manacor, Balearic Islands, Majorca, Spain; Chinese Academy of Sciences, CHINA

## Abstract

**Objective:**

To increase discriminatory accuracy (DA) for emergency cesarean sections (ECSs).

**Study design:**

We prospectively collected data on and studied all 6,157 births occurring in 2014 at four public hospitals located in three different autonomous communities of Spain. To identify risk factors (RFs) for ECS, we used likelihood ratios and logistic regression, fitted a classification tree (CTREE), and analyzed a random forest model (RFM). We used the areas under the receiver-operating-characteristic (ROC) curves (AUCs) to assess their DA.

**Results:**

The magnitude of the LR+ for all putative individual RFs and ORs in the logistic regression models was low to moderate. Except for parity, all putative RFs were positively associated with ECS, including hospital fixed-effects and night-shift delivery. The DA of all logistic models ranged from 0.74 to 0.81. The most relevant RFs (pH, induction, and previous C-section) in the CTREEs showed the highest ORs in the logistic models. The DA of the RFM and its most relevant interaction terms was even higher (AUC = 0.94; 95% CI: 0.93–0.95).

**Conclusion:**

Putative fetal, maternal, and contextual RFs alone fail to achieve reasonable DA for ECS. It is the combination of these RFs and the interactions between them at each hospital that make it possible to improve the DA for the type of delivery and tailor interventions through prediction to improve the appropriateness of ECS indications.

## Introduction

A worrisome issue in obstetrics is the longstanding increase in cesarean section rates, as well as the unjustified variations in these rates in clinical practice across public and private hospitals worldwide[1–3]. This is particularly important in the case of emergency (i.e., unscheduled) cesarean section (ECS) rates, assuming that the appropriateness of indications for scheduled C-sections is reasonably acceptable and much higher than that for ECSs [4–11]. Heterogeneity in clinical decision-making should always be investigated when unjustified variations are suspected. Knowing the fetal, maternal, and contextual factors that drive the decision to perform an ECS at each hospital is paramount to designing and implementing hospital-tailored interventions specifically aimed at improving the appropriateness of indications for ECSs in order to avoid unnecessary ECSs and the associated complications and costs [12–22].

Few current clinical guidelines and interventions target these objectives [23–29]. Those that do are neither based on a comprehensive set of proven fetal and maternal risk factors (RFs) with high discriminant accuracy (DA) nor designed to take into account contextual factors that have been shown to be associated with both an increased rate of unnecessary ECSs and unjustified variations in clinical practice. Furthermore, most RFs for ECSs should be considered putative, since they have mainly been selected by means of logistic regression models that usually lack information regarding both their goodness-of-fit and their DA [30–38]. Traditional measures of association alone are inappropriate to discriminate between who will suffer a given outcome and who will not. Therefore, interventions based on average risk estimates for people both exposed and unexposed to spurious RFs could be ineffective, inefficient, and even potentially harmful [12–22].

To our knowledge, very few studies have sought to improve the ability to predict which women are at higher risk of ECS. Those that do are limited to nulliparas, include only a few of the putative RFs, and report no measures of either calibration or DA of the statistical models developed [30–38]. Our objective is not to build an explanatory model of the decisions to perform an ECS, but to increase the predictive accuracy regarding this type of delivery in order to provide more validated information with the ultimate view to improving the appropriateness of indications for ECS and thus preventing unnecessary C-sections.

## Material and methods

The present study is part of a large multifaceted intervention intended to improve the appropriateness of the indications for ECSs in 22 public hospitals of the Spanish National Health Service launched by the Spanish Ministry of Health. Of those 22 participating hospitals, four (A, B, C, and D) were included in this study because their databases were the most reliable in terms of consistency and coverage to ensure that robust predictive models of ECSs could be built. In size and complexity, the obstetric services of these four hospitals belong to level II (out of III) of the Spanish National Hospital Catalogue. They can be considered representative of about 42% of all obstetrics services of the Spanish National Health Service that belong to this level, since they all have a very similar case mix, and attend pregnant women with similar obstetric risk.

The study population consisted of all 6,157 singleton births, with no exclusions, occurring in 2014 at four public hospitals located in three different autonomous communities of Spain. According to the Spanish National Institute of Statistics, these 6,157 births account for 1,5% of all yearly births in Spain (around 420,000/year). Hospitals A and B account for 26,5% of all births occurring yearly in the Autonomous Community of the Balearic Islands, Hospital C for 12,6% of those occurring in Galicia, and Hospital D for 2,0% of those occurring in Valencia (https://www.datosmacro.com/demografia/natalidad/espana-comunidades-autonomas).

Data were collected prospectively over 2014 and registered in a specifically designed database that included the fetal, maternal, and contextual independent variables (Table 1 and S1 Tables). All presentations were included in the analysis. All variables put forth in the medical literature as predictive variables (putative PFs) of the type of delivery were in principle considered in the study with few exceptions. Since birth weight is a post-delivery variable, it cannot be predictive of the type of delivery. The estimated preterm fetal weight could be considered a potential predictive variable. However, it is barely used given that its measurement is very imprecise (± 400 g) [1, 4, 6–10].

**Table 1:** Fetal, maternal, and contextual covariate definition and categorization.

Covariates	Covariate categorization
Age	< 35 or ≥ 35 years
Mother´s weight	> 90 kg
Mother´s height	≤ 1,5 μ
Mother´s Body Mass Index (BMI)	≤ 35 oρ > 35
Gestational age	≤ 36 weeks
Previous pregnancies	No (0) or Yes (≥ 1)
Smoker	Yes or No
Previous C-section	0 or ≥ 1
Comorbidity^1^	Yes (≥ 1) o No
Obstetric risk^2^	Yes or No
Labor induction^3^	Used or Not used
Intrapartum (scalp) pH	< 7.20 or ≥ 7.20
Night-shift delivery	Yes (C-section initiated between 9 p.m. and 4 a.m.) or No (initiated between 4 a.m. and 9 p.m.)
Fetus gender	Male (0) female (1)

^1^Defined as having one or more of the following comorbidities during pregnancy: anaemia, asthma, heart disease, coagulopathy, type I and II diabetes in pregnancy, treated autoimmune disease, treated epilepsy, treated mental disease, treated neurological disease, treated renal disease, hemiplegia, treated liver disease, treated hyper and hypotiroidism, HIV infection, chronic hypertension, idiopathic thrombocytopenic purpura, malignant tumor, hepatitis C and B virus, amniocentesis, corial biopsy, cordocentesis, cannabis, cocaine, heroin, other drugs, disseminated intravascular coagulation, colesthasys, corioamnionitis, pathological Doppler result, chronologically prolonged pregnancy, fetal death, stained amniotic fluid, pathological non-stress test, oligoamnios, small for gestational age, pre-eclampsia, premature rupture of membranes, prolonged pregnancy.

^2^Defined as the presence during pregnancy of one or more of the following factors that increase the chance of an adverse pregnancy outcome: cholestasis, chorioamnionitis, diabetes insulin and non-insulin dependent, chronologically prolonged pregnancy, multiple pregnancy, hellp syndrome, hypertension, isoimmunization in pregnancy, stained amniotic fluid, fetal malformation, uterine malformation, fetal malposition, myomectomy, oligoamnios, previous preterm labor, placenta praevia, plyhydramnios, preeclampsia, premature rupture of membranes, siphylis, toxoplasmosis, previous c-section, repeated abortions, previous miscarriages, anteparturm alteration of fetal wellbeing.

^3^All labors started by administering oxytocin or prostaglandins when indicated.

Unlike other predictive models published, we additionally included hospital fixed-effects and night-shift delivery as potentially predictive contextual independent variables. They are unobserved effects of hospital (contextual) characteristics that are not captured by any of the independent variables included in the models. They may be predictive of the type of delivery, account for a certain fraction of the medical variations (total variance) of ECSs often found in small area analysis, and modify the strength of the associations of the independent RFs and the type of delivery. They are not explanatory of the type of delivery, but their association with it may be indicative of different entrenched, difficult to measure clinical practices across hospitals that are likely to influence the decision regarding the type of delivery and therefore they warrant further investigation. Night-shift delivery was also included as an additional potentially predictive contextual independent variable, since it has been shown to be both a good predictor of the delivery mode, and an appropriate instrumental variable to infer causal associations between the average treatment effect of non-medically indicated cesarean sections (compared with vaginal delivery) on newborn´s health outcomes [39].

Descriptive statistics were calculated for all fetal, maternal, and contextual variables. Scheduled, emergency, and overall (both scheduled and emergency) C-sections were estimated for the whole population and for each hospital with their corresponding 95% CI.

The first step in our analytical approach to identify RFs for ECS was to calculate the prevalence of each putative RF in the overall population and in mothers delivering both by vaginal birth and by ECS, as well as their 95% CI. We then estimated the prevalence ratios of each RF (by dividing the prevalence of the RF by the prevalence of ECS). Finally, we estimated the positive likelihood ratios (LR+) of each RF and their 95% CIs. (A LR+ >10 is considered high enough to rule in the outcome, 5–10 is considered moderate, and 2–5 is considered low [40–47].

The second step was to build a logistic regression model for each of the four hospitals included in the study (A, B, C, and D), as well as a logistic model for the overall sample to find out which fetal, maternal, and contextual RFs (independent variables) were associated with the outcome (delivery type: vaginal or ECS), as well as the strength of the associations found. Model specification was performed based on stepwise top-bottom variable selection, and taking into consideration the clinical relevance of each variable. Crude and adjusted ORs were obtained, as well as their 95% CIs. The models’ goodness-of-fit was compared by means of the -2log-likelihood ratios and the Akaike information criterion (AIC). Their DA was assessed through their areas under the receiver-operating-characteristic (ROC) curves (AUCs) along with their 95% CI.

We then fitted a classification tree (CTREE or conditionally unbiased inference classification tree), a relatively new and useful predictive technique for studying RFs and outcomes based on the unbiased recursive splitting of the study population sample into subgroups according to the independent variables [48]. The underlying mathematical algorithm chooses which independent to split, their discriminatory value, and the order in which the splitting occurs. Outcome discrimination can thus be maximized at each step, making it possible to account for complex relationships between variables and their interactions and preventing both over-fitting and biased variable selection. The process develops a hierarchical tree structure that enables such simultaneous analyses and presents them in a clinically useful format [48–50].

Unlike CART models, CTREE can handle datasets with both categorical and numerical variables without producing biased splits, and the interpretation of both odds ratios and likelihood ratios is straightforward. Therefore, we used dichotomous variables to enable comparisons with other published studies despite a small potential loss of information. All births were included in the analysis, and anonymity was preserved. A database was constructed by two computer engineers, who also managed the transfer of data. Database quality was periodically audited and was considered reliable in terms of consistency, coverage, and agreement. The database is available upon request. The Spanish Ministry of Health approved this study under the Strategy for Assistance at Normal Childbirth in the National Health System (PI/01445).

We also developed a random forest model (RFM) that fits *n* classification trees by randomly selecting predictors for each tree. CTREE was used as the base learner, and 500 different trees were created by bootstrapping, rendering more accurate predictions than a single tree analysis. This algorithm allows to estimate the relative importance of each independent variable in the model (i.e. the contribution of each independent variable to the predictive power of the random forest). The methodology to compute relative importance of each variable (known as conditional permutation importance), and more information regarding CART, CTREE, and RFM can be found elsewhere [48–50]. We also compared the models’ discriminatory performance by means of their corresponding ROC curves. Goodness-of-fit analysis across the abovementioned models was performed using in-sample (n = 6,157) data with ROC curves. The statistical analyses were performed using R Statistical Software (Foundation for Statistical Computing, Vienna, Austria) [49, 50].

## Results

ECS rates varied from 8 to 15% across hospitals, whereas overall C-section rates were higher (12–21%) (Table 2). Descriptive population statistics are shown in Table 3. Mothers delivering by ECS were slightly older, had higher BMIs and weight, were more likely to have had a previous C-section, had more comorbidity, presented greater obstetric risk, more often underwent labor induction and delivered during the night shift, and had a slightly lower gestational age, and intrapartum (scalp) pH than those who had eutocic deliveries. No differences were found regarding smoking during pregnancy (Table 4).

**Table 2:** Emergency and overall (scheduled and emergency) cesarean rates by hospital.

	Number	Emergency rate (%)	95% CI	Overall rate (%)	95% CI
Hosp. A	1,923	8	7–9	14	13–15
Hosp. B	893	9	8–10	12	11–13
Hosp. C	2,458	15	14–16	21	20–22
Hosp. D	883	11	10–12	15	14–16
Total	6,157	11	11	17	17

**Table 3:** Descriptive population statistics.

Numeric Variables	Mean	Std. Dev
Mother´s age (years)	31.89	5.41
Mother´s weight (kg)	66	13,7
Mother´s height (m)	1.62	0.06
Previous pregnancies (No.)	1.23	1.25
Gestational age (weeks)	39.2	1.78
Categorical Variables	Percentage	Number
Smoker (Yes, No)	12.2	6,157
Previous C-section (Yes, No)	11.3	6,157
Comorbidity (Yes, No)	17.4	6,157
Obstetric risk (Yes, No)	40.6	6,157
Labor induction (Yes, No)	22.7	6,157
Scalp pH < 7.20	9.3	6,157
Night-shift delivery (Yes, No)	45.3	6,157
Fetus gender (male)	52.1	6,157

**Table 4:** Distribution of fetal, maternal, and contextual variables by delivery type.

	Mean		
Independent variables	Vaginal birth	Emergency C-sections	p-value
Age (years)	31.46	32.83	<0.001
Weight (kg)	65.7	67.9	<0.001
Height (m)	1.63	1.61	<0.001
BMI	23.96	26.66	<0.001
Gestational age (weeks)	39.3	38.8	<0.001
Fetus gender (%)	51.5	55.3	0.065
Previous pregnancies (mean)	1.125	1.257	<0.001
Smoker (%)	11.9	13.4	0.256
Previous C-sections (%)	10.1	22.4	<0.001
Comorbidity (%)	17	25	0.014
Obstetric risk (%)	35	58	<0.001
Labor induction (%)	20	43	<0.001
Intrapartum pH	7.296	7.245	<0.001
Night-shift delivery (%)	44	55	<0.001

The prevalence of the putative RFs for ECS in the overall population, as well as in eutocic and ECS deliveries, is shown in Table 5. In the overall population, the RFs with the highest prevalence (over 40%) were previous pregnancies, night delivery, BMI ≥ 25, and obstetric risk. The prevalence of all RFs except smoking and parity was higher in women delivering by ECS than in those with eutocic deliveries according to their 95% CI. All prevalence ratios were 6% or lower, and the LR+ of all individual RFs were low (4.14 or lower).

**Table 5:** Prevalence ratios and positive likelihood ratios of the putative risk factors for emergency C-sections.

	Overall prevalence	95% CI	Prevalence eutocic deliveries	95% CI	Prevalence emergency C-sections	95% CI	Prevalence ratio	LR+	95% CI
Smoker	12	12,12	12	12,12	13	12,14	1.104	1.08	1–1.16
Previous C-section	11	11,11	10	10,10	22	20–24	1.028	2.2	2–2.4
Comorbidity	17	17–17	17	16–18	25	23–27	1.585	1.47	1.38–1.56
Obstetric risk	41	40–42	38	37–39	58	56–60	3.69	1.52	1.47–1.57
Previous pregnancies	68	67–69	69	68–70	68	66–70	6.221	0.98	0.95–1.01
Induction	23	22–24	20	19–21	43	41–45	2.062	2.15	2.05–2.25
Scalp pH	9	9,9	7	7,7	29	27–31	2.636	4.14	3.85–4.42
Night-shift delivery	45	44–46	45	44–46	55	53–57	4.114	1.22	1.17–1.26
Mother´s weight (> 90kg)	3	3,3	5	5,6	9	8,10	1.08	1.03	1.01–1.06
Mother´s height (< 1.50 m)	3	3,3	3	3,3	5	4,6	1.14	1.03	1.01–1.04
Gestational age (≤ 36 weeks)	6	5,6	5	4,6	15	13–17	1.22	1.1	1.06–1.13
BMI ≥ 25	41	40–42	37	35–39	51	49–53	3.611	1.37	1.32–1.43
Age ≥ 35	27	26–28	26	25–27	34	32–36	2.441	1.3	1.23–1.38

The gender of the fetus was neither associated with the type of delivery nor improved either the calibration (-2 log likelihood ratios, AIC) or the discriminant accuracy (C statistic) of the final models. Therefore, it was excluded from the final logistic models. BMI was finally included since it did not make any difference to include height and weight separately or BMI in terms of both the calibration (AIC) and the discriminant accuracy (C statistic) of the models. We did choose the most parsimonious models as the final ones. Gestational age was also excluded from the final logistic models due to its high collinearity with the rest of the independent variables that remained in the model for each hospital, and because its inclusion led to biased intercept estimates of these logistic models.

According to the final logistic regression model for the overall population (Table 6), all RFs except for the number of previous pregnancies were positively associated with ECS. The strongest associations were those found for scalp pH (OR = 5.56), Hospital C (OR = 2.69), induction (OR = 2.32), and previous ECS (OR = 2.28). The remaining ORs were lower than 1.5, although the lower limits of their 95% CI were greater than 1.0. The only inverse association found was that between parity and ECS (OR = 0.87). With regard to the contextual variables, hospital fixed-effects and night-shift delivery were also positively associated with ECS. The strongest association was found with Hospital C, what is consistent with its substantial relative importance found in the random forest (Table 7).

**Table 6:** Logistic regression models to assess the association between the putative risk factors and type of delivery for the overall population and the four hospitals.

	4 Hospitals	Hosp. A	Hosp. B	Hosp. C	Hosp. D
Hospital A	1.05 (0.74–1.36)				
Hospital C	2.67*** (2.38–2.96)				
Hospital D	1.44*** (1.09–1.78)				
Age	1.02*** (1.01–1.04)	1.04** (1.01–1.08)	1.05** (1.01–1.10)	1.02 (0.99–1.04)	1.03 (0.99–1.08)
BMI	1.03*** (1.02–1.05)	1.04*** (1.01–1.087)	1.01 (0.96–1.10)	1.03*** (1.01–1.09)	1.04 (0.99–1.08)
Smoker	1.230(0.98–1.48)	1.56* (1.05–2.07)	0.92 (0.13–1.70)	1.32 (0.95–1.67)	0.97 (0.35–1.59)
Prev. ECS	2.28*** (2.04–2.51)	3.77*** (3.25–4.29)	3.06*** (2.43–3.69)	1.94*** (1.99–2.29)	2.32*** (1.70–2.95)
Comorbidity	1.21* (1.00–1.42)	1.41 (0.79–2.04)	2.43** (1.70–3.16)	1.05 (0.79–1.31)	1.34 (0.77–1.91)
Obstetric risk	1.57*** (1.38,1.766)	0.95 (0.54–1.37)	2.32*** (1.72–2.9)	2.07*** (1.81–2.33)	0.90 (0.34–1.47)
No. pregnancies	0.87*** (0.79–0.94)	0.75*** (0.57–0.93)	0.95 (0.73–1.16)	0.79*** (0.67–0.91)	1.27** (1.09–1.45)
Induction	2.23*** (2.14–2.50)	3.18*** (2.78–3.59)	1.72** (1.19–2.25)	2.14*** (1.87–2.40)	2.26*** (1.79–2.73)
Scalp pH	5.56*** (5.35–5.78)	5.24*** (4.81–5.66)	4.54*** (3.98–5.9)	5.69*** (5.31–6.07)	7.17*** (6.69–7.65)
Night-shift delivery	1.49*** (1.32–1.66)	1.40* (1.03–1.77)	1.11 (0.60–1.61)	1.78*** (1.54–2.02)	0.93 (0.47–1.39)
Observations	6,157	1,923	893	2,458	883
Log Likelihood	-1,843.93	-425.61	-219.91	-912.01	-255.236
AIC	3,715.86	873.21	461.82	1,846.04	532.47
AUC	0.7781	0.81	0.7942	0.7477	0.79
CI. AUC 95%	(0.76–0.7962)	(0.7784–0.8513)	(0.7393–0.849)	(0.7211–0.7743)	(0.7382–0.8418)

Note:

* p < 0.1,

** p < 0.05,

*** p < 0.01, 95% CI in parenthesis, AIC = Akaike Information Criterion, AUC = Area Under the Curve.

**Table 7:** Relative importance of each putative risk factor for type of delivery according to the random forest.

Variable	Relative importance
Scalp pH	100
Previous C-section	76.712
Induction	31.755
Hosp. C	29.895
BMI	27.854
Hosp. A	20.03
Obs. risk	11.635
Age	9.002
Pregnancies	4.901
Hosp. D	3.709
Smoker	3.194

The strength of the positive associations was relatively similar in the models for each of the four hospitals and in the model for the overall population. Although pH, induction, and previous ECS appear to be the RFs with the highest ORs, and age and BMI those with the lowest, their relative magnitude at each hospital varied slightly, except for pH, which was substantially higher at one hospital (OR = 7.17). Parity was positively associated with ECS at only one hospital, whereas obstetric risk was positively associated with it at only two.

The logistic model for the overall population and those for each hospital fit the data well, as indicated by both the -2log-likelihood ratio and the Akaike criterion. The goodness-of-fit of the population model increased notably when hospital fixed-effects were included. The DA of all five models was notably high, with AUCs ranging from 0.74 to 0.81 (Table 6).

**Fig 1:**
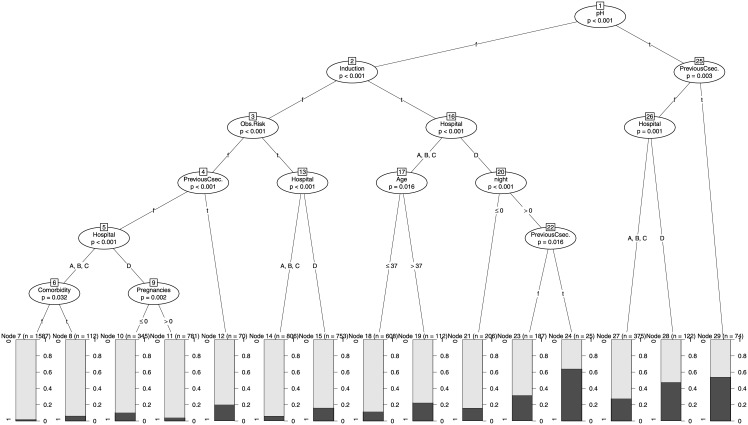


Of the two recursive partitioning models (CTREE and Random Forest), CTREE was used as the base learner for the Random Forest algorithm (n = 500). Fig 1 depicts the tree structure of the trained CTREE. The first split (p < 0.001) is scalp pH, followed by labor induction and previous ECS, for pH ≥ 7.20 and pH < 7.20 respectively, meaning that if the pH ≥ 7.20, the next split is birth induction (p < 0.001), whereas if the pH < 7.20, the next split is previous ECS (p = 0.003). The interpretation extends to the conditional nodes (splits) and leaves. By way of example of the meaning and utility of hospital effects, on the extreme right side of Fig 1 it can be seen that mothers whose fetuses had a scalp pH > 7.20 and had not had a previous ECS, in hospital D had a probability of almost 48% of having an ECS, whereas in the other hospitals (A, B, and C) this probability went down to 27%. The AUC mean value of the CTREE was 0.88 (95% CI: 0.84–0.92).

The RFM consisted of a set of *n* = 500 CTREEs with an optimal number of randomly selected variables = 2. Although random forest algorithms tend to be more of a black box in terms of their interpretation, their predictive power (AUC = 0.94; 95% CI: 0.93–0.95) provides reliable predictions even at an individual level. The relative variable importance of all variables included in the RFM is shown in Table 7. The three most relevant RFs (pH, induction, and previous ECS) also showed the strongest associations in the logistic models. Since the LR+ of all the interaction terms found in the RFM were lower than 10, as was the case for the individual RFs (Table 5), they failed to rule in the type of delivery.

## Discussion

The strength of the associations between some putative RFs and ECS, their prevalence, their prevalence ratios, and their LR+ in the overall population were low to moderate, indicating, as in other studies, that single RFs alone offer only a low DA for most outcomes, such as ECS [40–47].

With the exception of scalp pH, the magnitude of the strength of these associations was low and similar across the four hospitals. Likewise, all were positive except for the number of pregnancies, which showed an inverse association. Heterogeneity did not seem to play a relevant role in the study population solely on the basis of this initial analysis. Moreover, only the number of pregnancies seemed to increase the odds of a vaginal delivery, as would be expected.

In the final logistic model for the overall population both contextual variables (hospital fixed-effects and night-shift delivery) were positively associated with ECS and increased goodness-of-fit. These variables were associated with higher ECS rates and may thus favor the indication of ECS over vaginal deliveries. Regardless of maternal and fetal characteristics, and as indicated in a number of studies, different entrenched practices across hospitals seem to influence the decision regarding delivery type, similar to how physicians’ desire for night-time leisure influences the decision to perform an ECS at the start of the night shift [4–11, 39].

No single 100% accurate predictive model of the type of delivery has been published to date. In fact, only a few have been published all showing a low predictive and discriminant accuracy. All these contextual (hospital) factors that may contribute both to predict and explain variations in both the type of delivery and in the appropriateness of the c-section´s indications (as shown by the high variability of rates of c-sections in several published atlases of variations in medical practice) remain unobserved and unknown. The only available way to account for them is by including hospital fixed-effects in logistic models and in random forests as contextual variables (which are tantamount of the second level variables in multilevel analyses). Moreover, their inclusion in the models reduced the biases in the estimates of the measures of strength of the associations without resulting in overfitting, and increase their discriminant accuracy because they account for the abovementioned unobserved predictive factors [4–11, 39].

These results illustrate the usefulness of this analytic approach because they suggest that some hospital characteristics (i.e., method of payment and other incentives, physicians’ desire for night-time leisure, established non-evidence-based practices such as to perform a c-section to mothers having had a previous c-section) may explain unjustified variations and inappropriateness of some indications for c-sections that warrant further investigation.

Consequently, all fetal, maternal, and contextual factors alone failed to achieve a reasonable DA for ECS rates in different population subgroups at each hospital even after they were controlled for in these models. This is consistent with the well-known fact that the decision regarding the type of delivery hinges not only on different combinations of these RFs and the interactions between them, but also to some extent on variations across individual hospital practices and even individual clinicians’ practices. It can thus be the product of unjustified non-evidence-based clinical practices, which has long been shown in studies of variations in clinical practice with regard to CS using small area analysis [4–11].

Measures of association alone are insufficient to discriminate between those individuals who will develop a given outcome and those who will not (a strong association is not tantamount to high DA given that the false positive and false negative fractions of the population are low) [40–43]. It is the set of independent variables included in the final logistic models that could make it possible to achieve acceptable DA, as shown by their high AUC (0.75–0.81). To our knowledge, no logistic regression model published to date has achieved an AUC similar to those reported here.

The AUCs of the RFM (0.93–0.95) and the CTREE (0.84–0.92) offer a considerably improved additional analytical approach to the same issue due to the nature of their optimization algorithm, maximum likelihood for logistic and unbiased recursive partitioning for CTREE. Their incremental DA is notably higher than that of logistic models due to the unsupervised detection of interactions in the CTREE model and 500 such CTREEs in the RFM. The reasons for this improvement in DA are mainly twofold. First, it results from detecting associations and interactions among the combinations of RFs used in clinical decision-making regarding the type of delivery at each hospital that are not captured by logistic models. Second, the model also captures heterogeneity (the trees’ branches), among both the hospitals and the clinicians’ decision-making frameworks, that logistic models likewise cannot capture.

In terms of implications for clinical practice, we found some medically unjustified differences in ECS rates for hospital D compared to the other hospitals, e.g., in induced births between 11 p.m. and 3 a.m. in which the scalp pH was above 7.20 (nodes 2, 16, and 20). Moreover, in the subgroups of deliveries with pH above 7.20 and at least one previous C-section (nodes 25 and 26), the ECS rates climbed to 50% and almost 60%, respectively. The utility of these results lies in that, despite they are neither explanatory not confirmatory, they suggest potential sources of inappropriate ECSs in Hospital D (contextual factors) that should be further investigated (i.e., changes in payment methods, lack of updated clinical guidelines, lack of utilization management, demand side issues).

One of the main limitations of this study is that only 4 out 22 obstetrics services were included as explained in the Introduction. These four hospitals could be considered representative of up the 42% of hospitals within the Spanish National Health Service in terms of obstetric case mix, obstetric risk, and number of births and CS rates. However, it is to be expected that studies intended to build a predictive model for the type of delivery fail to have a high external validity with regard to the specific RFs for ECS. As already noted, it is the combination of RFs (fetal, maternal, and contextual) at each particular hospital and the interactions between them what makes it possible to improve the DA for the type of delivery. The more the clinical practice varies across centers and clinicians, the more different RF-combination subgroups can be expected to appear in the CTREES given their higher ability to capturing them; hence, the more hospital-specific the combination of RFs and interactions between them yielding the highest DA will be. Given that we performed a 10-fold cross-validation using randomly allocated 90/10% training/test sample sizes, the chances of the RFM being overfitted and the AUCs being overestimated are very low.

Another limitation of the study is that scalp pH is a very proximate measure likely linked to fetal distress, so it is not a surprise that it is highly predictive. We did not include cord pH because it is a post-delivery endpoint and as such cannot be considered a predictive variable of the type of delivery. We could agree that scalp pH is linked to fetal distress and can be highly predictive. However, we have included it in the models as a predictive variable for several reasons: i) scalp pH is an intrapartum variable, not a final endpoint. Variations in the cut-off points actually used in clinical practice may explain both variations in the diagnosis of fetal distress, and in the fraction of appropriate and inappropriate indications for ECSs across hospitals (as it have been shown is studies of the appropriateness of the different types of emergency ECSs indications, in this particular case, fetal distress); ii) it has also been shown that both the clinical management of intrapartum (scalp) pH and thus of fetal distress varies across hospitals, and that it accounts for a considerable fraction of inappropriateness of ECSs for this specific indication, what could make scalp pH a predictive variable for some but not all ECSs; and iii) tenfold cross validation performed in the CTREE model prevented from obtaining overfitted estimates when including this variable.

Therefore, this study’s main contribution is that the information provided by the combination of logistic regressions and CTREES can provide more accurate information than either method alone to help clinicians and managers find the sources of heterogeneity and unjustified variations in ECSs, design and implement hospital-tailored interventions intended to improve the appropriateness of their indications, and reduce unnecessary ECS and their avoidable complications and costs. This comprehensive and complementary statistical methodology, combined with robust data collection and audit processes, makes it possible to analyze an intricate medical decision-making problem with higher discriminant capacity than previous studies.

In conclusion, fetal, maternal, and contextual factors alone fail to achieve a reasonable discriminatory accuracy for type of cesarean delivery. We have met our objective by simultaneously considering these factors at each particular hospital by using both logistic regressions and the CTREES for the following reasons. First, this analytical strategy has improved the final discriminatory accuracy of the models for the type of delivery compared with that of the predictive models published to date. Second, the discriminatory accuracy of these models has been validated in our study by means of ten-fold cross-validation. Third, the results allow for further investigating sources of variability and inappropriateness of ECSs. Finally, based on this information, they also allow for tailoring hospital-specific interventions intended to discriminatory accuracy improve the appropriateness of indications for ECS.

## Supporting information

S1 Tables Database that includes the fetal, maternal, and contextual independent variables of hospitals A, B, C, and D. (XLSX)
